# The Effect of Divinylbenzene on the Structure and Properties of Polyethylene Films with Related Radiation Chemical Grafted Polystyrene and Sulfocationite Membranes

**DOI:** 10.3390/membranes13060587

**Published:** 2023-06-07

**Authors:** Anatoly E. Chalykh, Ramil R. Khasbiullin, Ali D. Aliev, Vladimir V. Matveev, Vladimir K. Gerasimov, Nikita A. Slesarenko, Irina A. Avilova, Vitaly I. Volkov, Vladimir A. Tverskoy

**Affiliations:** 1 Frumkin Institute of Physical Chemistry and Electrochemistry of Russian Academy of Sciences (RAS), Leninsky Pr. 31-4, 119071 Moscow, Russia; chalykh@mail.ru (A.E.C.); khasbiullin@techno-poisk.ru (R.R.K.); ali_aliev1948@mail.ru (A.D.A.); matveev46@yandex.ru (V.V.M.); vladger@mail.ru (V.K.G.); 2 Federal Research Center of Problems of Chemical Physics and Medicinal Chemistry of Russian Academy of Sciences (RAS), 142432 Chernogolovka, Russia; wownik007@mail.ru (N.A.S.); irkaavka@gmail.com (I.A.A.); vitwolf@mail.ru (V.I.V.); 3 Lomonosov Institute of Fine Chemical Technologies, MIREA—Russian Technological University, 119454 Moscow, Russia

**Keywords:** copolymerizaion, diffusion, divinylbenzene, graft polymerization, irradiation polymerization, morphology, polyethylene, styrene, sulfocationite membranes

## Abstract

In the present work, the effect of divinylbenzene (DVB) on the kinetics of post-radiation chemical graft polymerization styrene (St) on polyethylene (PE) film and its structural and morphological features were investigated. It has been found that the dependence of the degree of polystyrene (PS) grafting on the DVB concentration in the solution is extreme. An increase in the rate of graft polymerization at low concentrations of DVB in the solution is associated with a decrease in the mobility of the growing chains of PS. A decrease in the rate of graft polymerization at high concentrations of DVB is associated with a decrease in the rate of diffusion of St and iron(II) ions in the cross-linked network structure of macromolecules of graft PS. A comparative analysis of the IR transmission and multiple attenuated total internal reflection spectra of the films with graft PS shows that graft polymerization of St in the presence of DVB leads to the enrichment of the film surface layers in PS. These results have been confirmed by the data on the distribution of sulfur in these films after sulfonation. The micrographs of the surface of the grafted films show the formation of cross-linked local microphases of PS with fixed interfaces.

## 1. Introduction

Synthetic membranes are heterogeneous solid phase barriers between two phases. The transport of molecular/ionic species of liquids or gases is carried out by contact with their surfaces under the influence of a driving force. Synthetic membranes can selectively transport one species of liquid or gas over another (selectivity or degree of separation) or regulate the transport of different species at different controlled rates (permeability or flux). The transport of molecular/ionic species across membranes is driven by differences in the size, shape, chemical properties, or electrical charge of the components of the mixture being separated. The separation properties of a membrane are determined by both the chemical and physical nature of the membrane materials, as well as the method of preparation [1].

Among the various methods of ionite membrane production, grafting polymerization occupies a special place, which makes it possible to regulate their physicochemical and operational properties within a wide range [2,3,4]. The nature of the monomer, conditions of graft polymerization, and degree of grafting make it possible to modify the surface layer of the polymer material, its volume, and the uniform or gradient distribution of the graft polymer over the thickness of the polymer matrix [4,5]. There are various methods of graft polymerization [6]: “direct” graft; “post effect” graft under irradiation of polymer in a vacuum, inert atmosphere, or in air; and “post effect” radiation graft polymerization under irradiation of polymer in air. The latter method is simple and highly reproducible. In this case, the polymerization process is initiated by the alkoxy radicals generated during the decomposition of hydro- and diperoxides. As peroxide-reducing agents, to lower the polymerization temperature, salts of metals of variable valence are added to the monomer solution. As a rule, these are the iron(II) salts [5].

In most cases, during the synthesis of such membranes, graft polymerization of St is carried out on films of polyolefins or polyfluoroolefins, followed by the chemical modification of graft polystyrene (PS) and the production of cation-exchange membranes. Such sulfocationite membranes obtained by the sulfonation of PE films with grafted PS have high selectivity when separating mixtures of ethylene with ethane [7,8] and butenes with butane [9]. They are efficient as separation membranes of fuel cells [10].

In the earlier works of Chalykh et al. [11], we studied the effect of various parameters of post-radiation chemically grafted polymerization of St on a PE film on the kinetics of graft polymerization, the distribution of grafted PS along the cross-section of the PE film, thermochemical parameters and morphology of the phases of PS and PE, and energy characteristics of the surface of the grafted polymer. It was shown in [12] that the addition of small amounts (~1 vol. %) of DVB led to an increase in the degree of grafting of PS during radiation graft polymerization “under the beam” of St on a PE film.

Of particular interest is the diffusion of water molecules, which determines the ionic conductivity [13]. The diffusion coefficients of water in ion-exchange membranes measured by pulsed field gradient ^1^H NMR (PFG NMR) demonstrate two types of water molecules in the films with low DVB contents.

The purpose of this work is to study the effect of DVB on the post-radiation chemical graft polymerization of St on the PE film, the distribution of grafted PS over the thickness of this film, the morphology of these films, and the properties of related sulfocationite membranes.

## 2. Experimental

### 2.1. Materials and Reagents

The following materials and reagents were used: high-pressure PE film with a density of 0.92 g/cm^3^ and a thickness of 20 µm, St (Sibreaktiv, pure grade); DVB (Aldrich, St. Louis, MO, USA, technical grade, 55%); potassium hydroxide (Khimmed, Moscow, Russia, analytical grade); iron(II) sulfate heptahydrate (Khimmed, chemical purity grade); methanol (Khimmed, reagent grade).

St was purified from the inhibitor by successive treatment with an aqueous solution of potassium hydroxide and deionized water, drying, and distillation under vacuum to withdraw the boiling fraction at 40 °C at a residual pressure of 20 mmHg.

### 2.2. Preparation of Membranes

The PE film was irradiated in air at room temperature under a ^60^Co γ-radiation source with a radiation dose of 50 KGy and power of 5.2 Gy/s. The grafting copolymerization of St and DVB was carried out in a mixture of a methanol solution of 50 vol. % St and DVB and 50 vol. % methanol at the boiling point. The grafting solution contained 2 g/L iron (II) sulfate as a homopolymerization inhibitor and a co-initiator.

The degree of grafting (Δ*p*, %) of the copolymer of St and DVB was determined using Equation (1):

(1) $$\Delta p=\frac{m_{1}-m_{0}}{m_{0}}\cdot 100$$

where *m*_0_ and *m*_1_ are the film weights (in g) before and after the grafting copolymerization stage, respectively.

Sulfonation of the films was carried out in concentrated (96 wt. %) sulfuric acid at a temperature of 98 °C. At the end of the process, the membranes were washed with decreasing concentrations of sulfuric acid solutions and distilled water.

### 2.3. Physicochemical Techniques

IR transmission and multiple attenuated total internal reflection spectra were recorded using a Perkin Elmer-580 spectrophotometer (Stockholm, Sweden). Multiple attenuated total internal reflection spectra were recorded using a KRS-5 crystal with an incidence angle of 45° and a film scanning depth of ~5 µm.

Differential scanning calorimetry (DSC) was used to record thermal effects accompanying the melting (crystallization) and glass transition of the graft copolymer under the conditions of programmed temperature changes. The heating rate varied from 4 to 50 deg/min. Measurements were performed on a DSC 204 F1 Phoenix (Netzsch, Selb, Germany) in the temperature range from 25 to 150 °C in an Ar atmosphere with a flow rate of 60 mL/min. All experiments were performed using samples weighing at least 2 mg. For automatic processing of measurement results, we used the Proteus Analysis software (Version 6.1.0) in which the glass-transition temperature value *T_g_* was defined as an inflection point of the Δ*C_p_(T)* curve, and the melting point value *T_m_* was defined as a maximum (peak) point. The surface area of the peak bounded by the DSC curve and zero baseline was assumed to be equal to a change in the melting enthalpy (Δ*H_m_*). To determine the glass transition temperature, we also used the tangent method [14], which was applied to the left, right, and middle parts of the heat capacity step Δ*C_p_(T).*

The degree of crystallinity of the studied sample was determined using the following equation:

(2) $$\alpha =\frac{\Delta H}{\Delta H_{100\%}}$$

where Δ*H* is the melting enthalpy of the studied sample; Δ*H*_100%_ is the melting enthalpy for the completely crystalline polymer; and Δ*H*_100%_ = 293 J/g [15].

The structural and morphological characteristics of the graft copolymers were studied using transmission electron microscopy, scanning electron microscopy, and X-ray microanalysis. In the first case, the outer surface of the graft films was used as the object of the study, which was subjected to etching in high-frequency oxygen plasma in order to reveal the supramolecular structure. The oxygen pressure in the etching zone was 0.03 mm Hg, the electron energy was 2–3 eV, the etching time was 15–20 min, the generator power was 100 W, and the frequency was 10 MHz. The morphology of the etched surfaces was examined via the method of one-step carbon-platinum replicas using a TEM-301 transmission electron microscope (Amsterdam, The Netherlands) at an accelerating voltage of 80 keV.

In the cases of scanning microscopy and X-ray microanalysis, studies were carried out on cross-sections of the sulfated graft PE film obtained on an LKB cryo ultramicrotome. Surface analysis was performed using a JSM-U3 scanning electron microscope (JEOL, Tokyo, Japan) at an accelerating voltage of 5–7 keV equipped with an EDAX energy dispersive spectrometer (Hamburg, Germany).

The *o*-xylene absorption (Δ*m*, %) was determined according to Equation (3):

(3) $$\Delta m=\frac{m_{s}-m_{d}}{m_{d}}\cdot 100$$

where *m_s_* and *m_d_* are the film weights (in g) of the swollen and dry films, respectively.

The degree of swelling on the surface area (Δ*S*, %) in *o*-xylene was calculated from the surface area values of the film in the swollen state (*A_s_*) and in the dried state (*A_d_*) using Equation (4):

(4) $$\Delta S=\frac{A_{s}-A_{d}}{A_{d}}\cdot 100$$

The self-diffusion coefficients of water in the membranes with different DVB contents were measured for ^1^H with the pulsed field gradient technique at frequencies of 400.22 MHz. The measurements were carried out on a Bruker AVANCE-III-400 NMR spectrometer equipped with a diff60 gradient unit. A pulsed field gradient-stimulated echo sequence was used. Three 90° pulses produced a stimulated spin echo at time 2*τ* + *τ*_1_ (where *τ* is the time interval between the first and second 90° pulses, and *τ*_1_ is the interval between the second and third pulses). The magnetic field gradient pulses of amplitude *g* and duration *δ* were applied after the first and third 90° pulses. The gradient strength varied linearly in 32 steps within a range from 0.1 to 27 T/m. The integrated intensities of the spectral lines were used to obtain the dependence of the echo signal attenuation on *g*^2^ (diffusion decay) [16,17]. The evolution of the spin echo signal can be explained using the following equation:

(5) $$A(2\tau ,\tau_{1},g)=A(2\tau ,\tau_{1})\exp(-\gamma^{2}g^{2}\delta^{2}t_{d}D_{s})$$

where *γ* is the gyromagnetic ratio, Δ is the interval between the gradient pulses, *t_d_* = Δ − *δ*/3 is the diffusion time, and *D_s_* is the self-diffusion coefficient. *A*(2*τ*, *τ*_1_, 0) can be expressed using the equation

(6) $$A(2\tau ,\tau_{1},g)=\frac{A(0)}{2\exp\left(-\frac{2\tau }{T_{2}}-\frac{\tau_{1}}{T_{1}}\right)}$$

where *A*(0) is the signal intensity after the first radio frequency (RF) pulse; and *T*_1_ and *T*_2_ are the spin–lattice and spin–spin relaxation times, respectively. During the measurement of the echo signal evolution, *τ* and *τ*_1_ were fixed and only the dependence of *A* on *g* (diffusion decay) was analyzed.

The experimental diffusion decays were well approximated using Equation (5) by 2–3 orders of magnitude, and the self-diffusion coefficient measurement error was lower than 10%.

For NMR measurements, the membrane samples were dried by equilibration with P_2_O_5_ at room temperature after they were placed in a desiccator over water vapor until a constant weight was reached. Membrane plates 3 mm × 40 mm in size were inserted in a hermetically sealed NMR tube with an outer diameter of 5 mm.

## 3. Results

**Figure 1:**
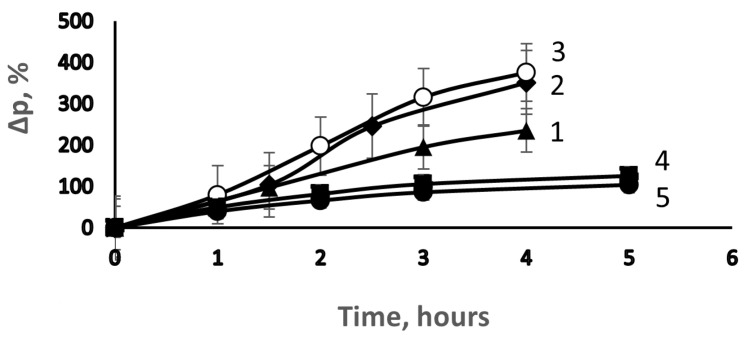
Effect of DVB on the kinetics of grafting polymerization of St. [DVB], vol. %: 1—0, 2—0.25, 3—0.5, 4—1.5, and 5—2.5.

Figure 1 shows the typical dependence of the degree of grafting of PS on time with a change in the concentration of DVB in the polymerization solution from 0 to 2.5 vol. %. It can be seen that at low concentrations (up to 0.5 vol. %), the addition of DVB leads to an increase in the initial rate of grafting polymerization. Upon reaching the degree of grafting ~70%, the gel effect (Trommsdorff effect) is observed. It should be noted that a similar increase in the degree of grafting of PS on PE in the presence of DVB was observed in [12] with direct grafting. In [14], it was also shown that the addition of DVB to St leads to an increase in the overall polymerization rate and the appearance of the gel effect. In our opinion, such an effect of DVB on the kinetics of St polymerization is due to the fact that the addition of DVB leads to the cross-linking of growing PS chains, a decrease in their translational mobility and, consequently, a decrease in the termination constant. In addition, an increase in the degree of grafting in the presence of DVB is possible due to the interaction of unvaccinated growing PS chains formed as a result of the chain transfer to the monomer and thermal polymerization of styrene with the unreacted double bond of DVB in the chain of the graft polymer.

**Figure 2:**
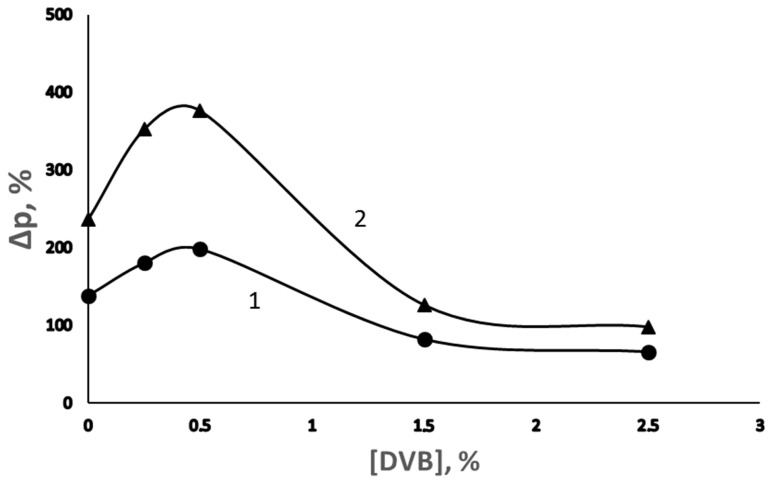
Dependence of the degree of grafting of PS on the PE film on the concentration of DVB in the grafting solution at a polymerization time of 2 (1) and 4 h (2).

The same figure shows that at the concentration of DVB above 0.5 vol. %, the initial rate of grafting polymerization is reduced. A possible reason for this effect is a decrease in the diffusion rate of the monomer and Fe^2+^ ions in the cross-linked network structure of the macromolecules of the grafted PS. The dependence of the degree of grafting on the concentration of DVB acquires a pronounced extreme character (Figure 2).

**Figure 3:**
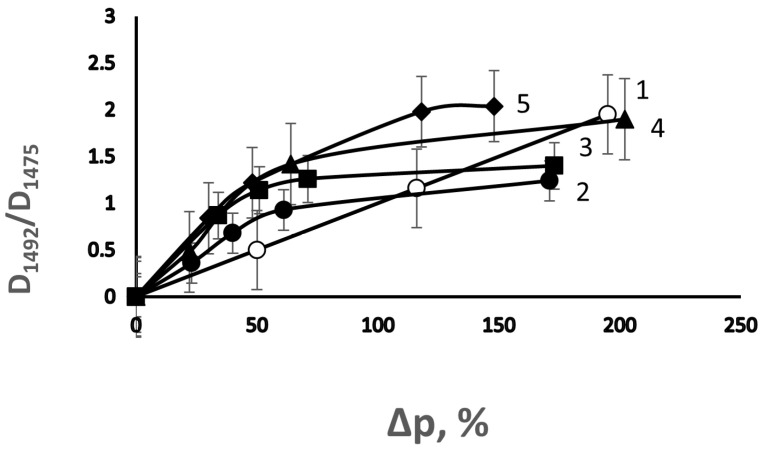
Effect of DVB on the dependence of the ratio of optical densities of the absorption bands at 1492 and 1475 cm^−1^ (D_1492_/D_1475_) in the transmission spectra (1) and multiple attenuated total internal reflection spectra (2–5) on the degree of grafting of PS on the PE film. [DVB], vol. %: 2—0; 3—0.25; 4—0.5; and 5—2.5.

Since the formation of the network limits monomer diffusion in the presence of DVB, the distribution of the grafted PS over the thickness of the PE film should depend on the concentration of DVB. This distribution was investigated by comparing the ratio of the optical densities of the bands at 1492 (stretching vibrations of the C-C bond in the phenyl ring of PS) and 1475 cm^−1^ (asymmetric bending vibrations of the C-H bond in PE) (D_1492_/D_1475_) [18] measured in the transmission and multiple attenuated total internal reflection spectra [18,19]. Indeed, as can be seen in Figure 3, an increase in the concentration of DVB in the polymerization solution leads to an increase in the D_1492_/D_1475_ ratio, which was measured using the multiple attenuated total internal reflection method; over this ratio, which was measured using the transmission method, the enrichment of the film surface in PS occurs. Interestingly, when the degree of grafting reaches ~100%, the concentrations of the grafted polymer on the surface and in the volume of the film are leveled, which indicates the course of the grafting process throughout the entire volume of the film in spite of the formation of network structures.

**Figure 4:**
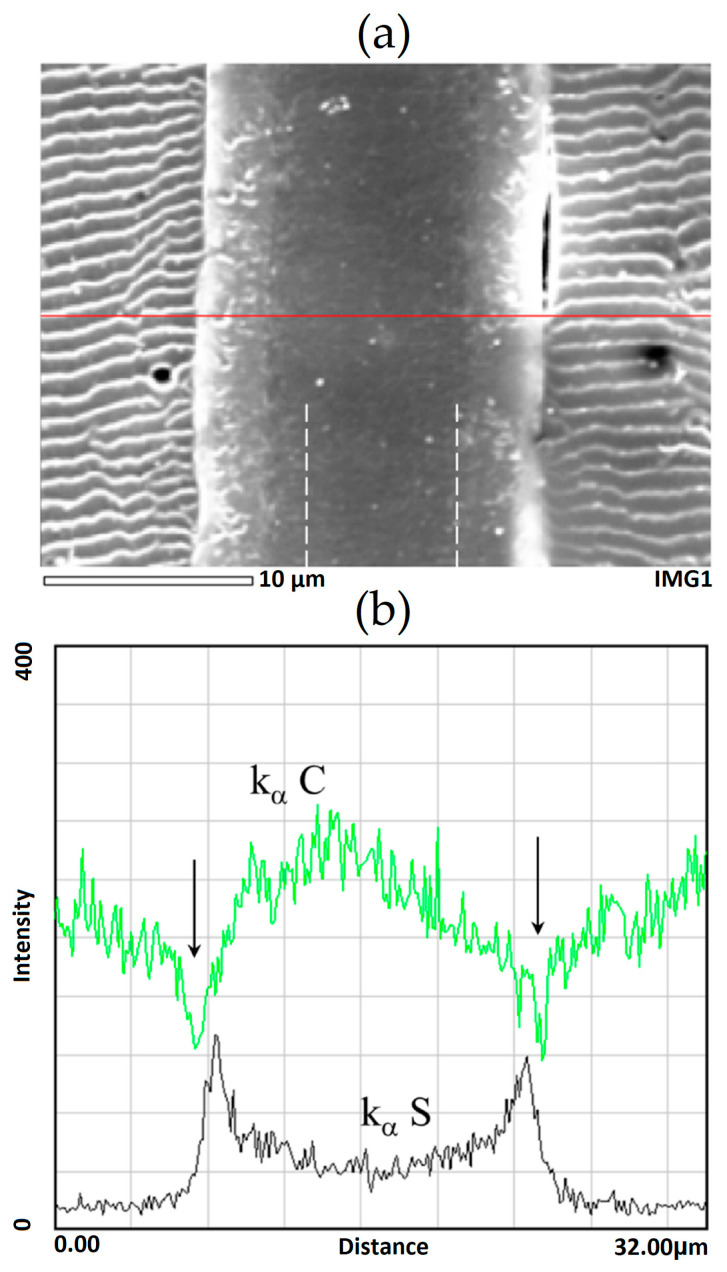
Microphotograph of the cross-section of the grafted film with Δ*p* = 93% ([DVB = 1 vol. %) in secondary electrons (**a**) and the profile of the characteristic radiation distribution of the K_α_S line (contrasted grafted film) and K_α_C line along the cross-section of the sample (**b**). The red line is the path taken by the elemental analysis probe. Dotted vertical lines is microphotograph of the cross-section film obtained in the second electrons. The arrows indicate the defective areas in the grafted layers.

Figure 4 shows a typical microphotograph of the cross-section film obtained in the second electrons (marked by dotted vertical lines) when grafted polymerization was carried out in the presence of DVB. It can be seen that the graft layer differs slightly in contrast to the central part. The distribution of the characteristic X-ray emission of the Kα sulfur and carbon lines shows a change in the PS concentration from the film surface layers to the center. The arrows indicate the defective areas in the grafted layers.

**Figure 5:**
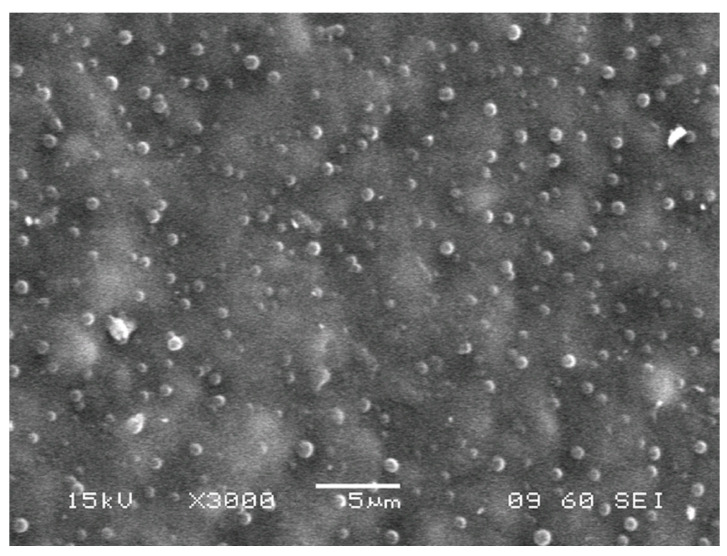
Micrograph of the surface of the grafted PE film obtained using a scanning electron microscope. Δ*p* = 67%, [DVB] = 0.5 vol. %.

**Figure 6:**
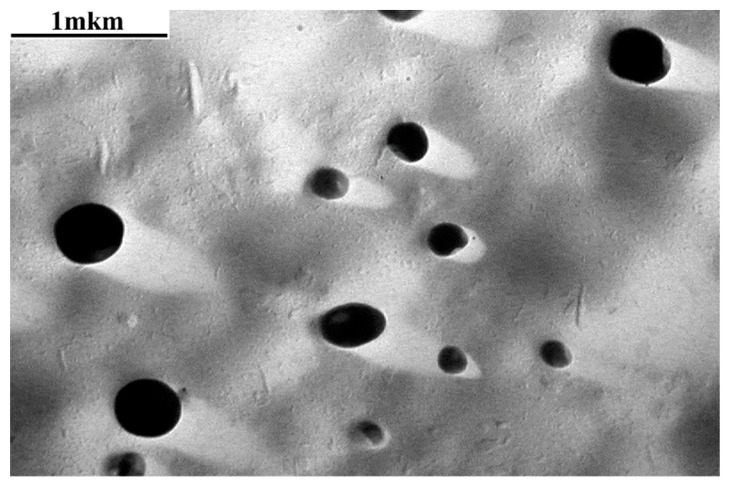
Micrograph of the surface of the grafted PE film obtained using a transmission electron microscope. Δ*p* = 67%, [DVB] = 0.5 vol. %.

The micrographs of the surface of grafted samples (Figure 5 and Figure 6) demonstrate the formation of spherical particles: cross-linked phases of PS, the average size of which varies in the range from 0.3 to 0.8 μm, and their concentration reaches ~5 vol. %. According to the local X-ray microanalysis data, the elemental composition of these phases does not differ from that of the composition of the dispersion medium; therefore, we assume that these dispersed phases belong to fragments of PS microgels cross-linked with DVB. These dispersed systems are observed in the films with both low (Δ*p* = 34%) and high (Δ*p* = 93%) degrees of grafting. Their concentration does not increase with an increasing degree of grafting. This suggests that DVB is consumed in the early stages of grafting polymerization. This is consistent with the fact that DVB is a more active monomer during its copolymerization with styrene [20].

The distribution of the grafted polymer over the thickness of the PE film can be determined based on the distribution of sulfur in the film after sulfonation.

**Figure 7:**
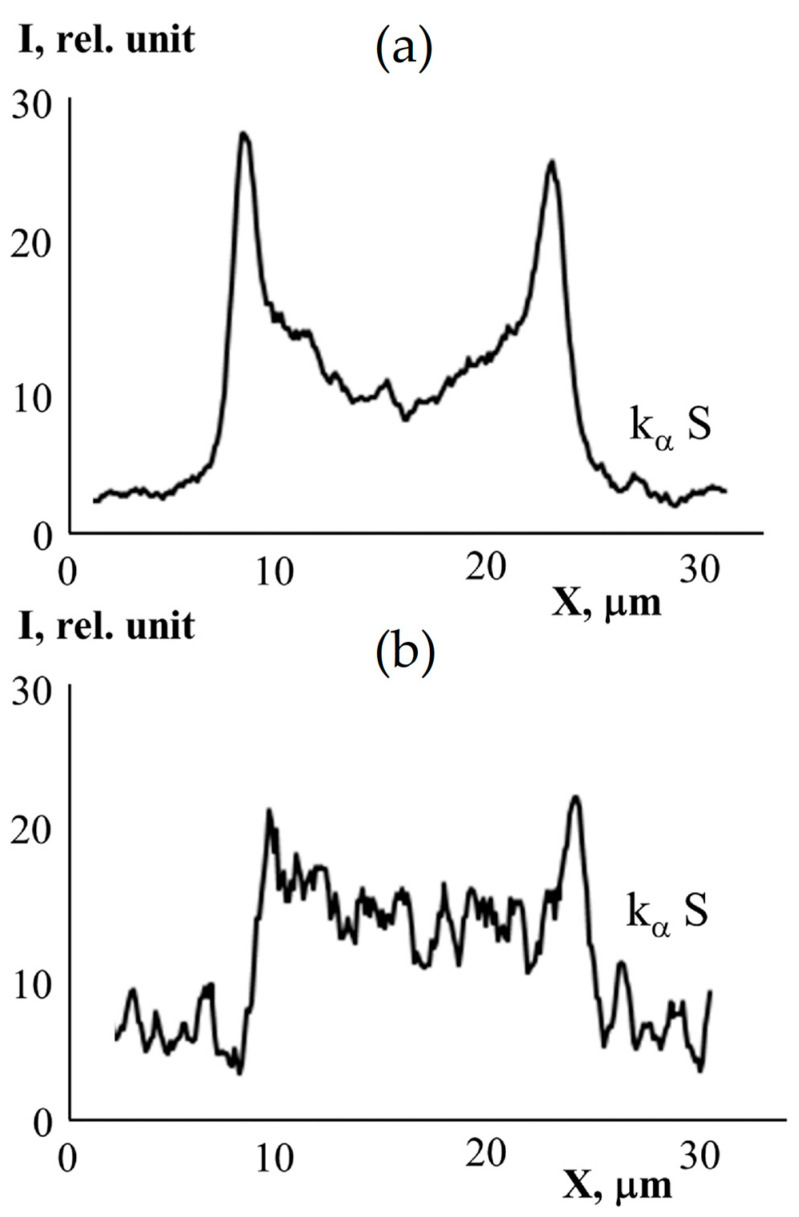
Distribution of sulfur over the cross-section of the PE film with grafted PS (Δ*p* = 90%) (**a**) and the film with grafted PS in the presence of DVB (Δ*p* = 67%, [DVB] = 0.5 vol. %) (**b**).

The graft polymerization of St on the PE film was carried out in [11,19] under the same conditions as in the present work. In these works, it is shown that the surface layers of the films are enriched with PS. A similar pattern of the distribution of PS over the thickness of the PE film was observed in this work (Figure 4 and Figure 7).

**Figure 8:**
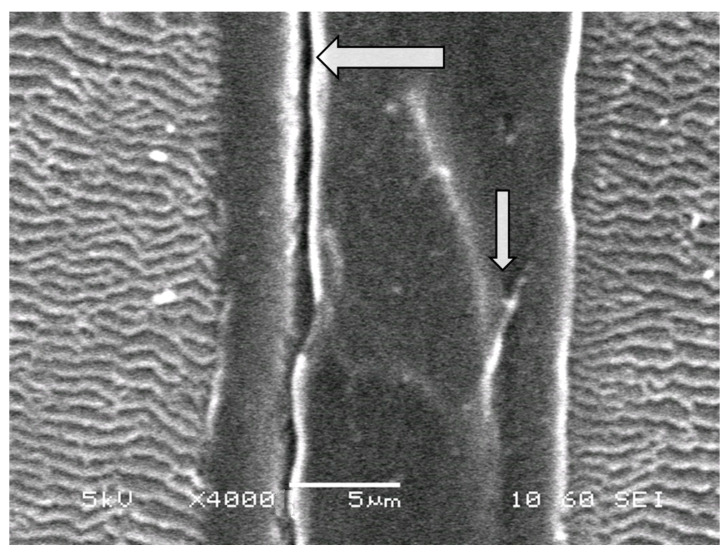
Defective areas on the cross-section of the PE film with grafted PS (Δ*p* = 74%) in the presence of DVB (2.5 vol. %). Arrows indicate defects in the grafted layers of PS.

The figure shows that the surface layers of the films are enriched with sulfur. The difference between the supramolecular organization of the sulfurized PE-PS-DVB film and the binary PE-PS system is the increased nonuniformity of the sulfur distribution, the presence in the volume and on the surface of microphase particles of cross-linked DVB having clearly fixed interfaces, and the formation of the contact zone of the grafted layer with the volume of the film of extended defects and cracks, arising as a result of the formation of internal stresses of thermochemical origin. (Figure 8).

**Figure 9:**
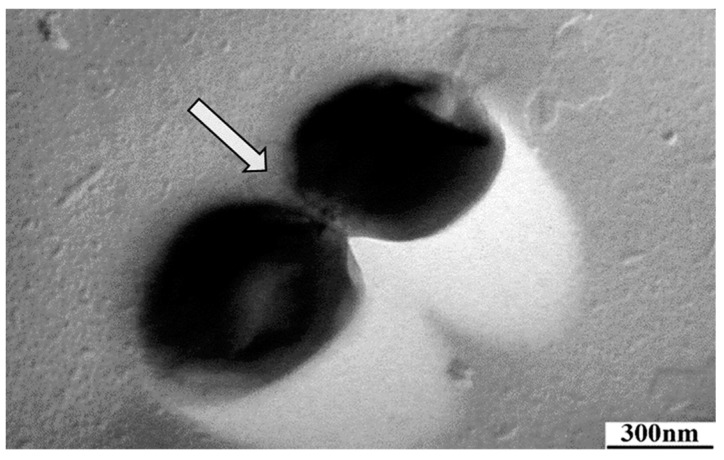
Coalescence of the phases of grafted PS in the presence of DVB in the PE matrix (Δ*p* = 74%, [DVB] = 0.5 vol. %). The arrow marks the contact zone of the particles.

**Figure 10:**
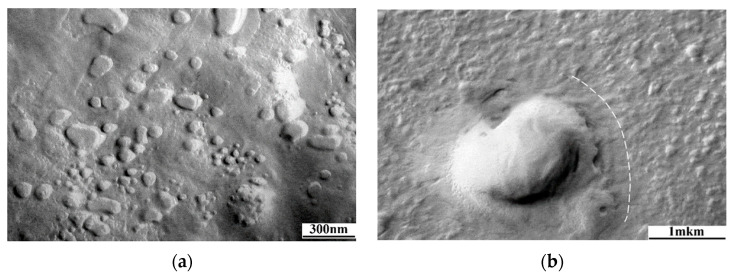
(**a**) Phase distribution of the grafted PS in the volume of the PE film (obtained in the cross-section of the membrane); (**b**) the phase particle of the cross-linked PS in the PE matrix (Δ*p* = 74%, [DVB] = 0.5 vol. %). Dotted line indicates the particles of the PE lamella bonded to the surface.

In our opinion, the nonuniformities of the sulfur distribution are the result of structural and morphological heterogeneities in the organization of the membrane films that occur during the graft polymerization of the monomer, and the participation of polymerization products in the formation of spatially cross-linked structures of macromolecules of the grafted polymer when their fragments lose translational mobility. This induces the microgel particles to spread and coalesce in their contact zone (Figure 9). Nevertheless, the graft polymerization process continues, and the final stage leads to a more uniform distribution of the grafted polymer over the entire thickness of the film (Figure 7b and Figure 10).

**Figure 11:**
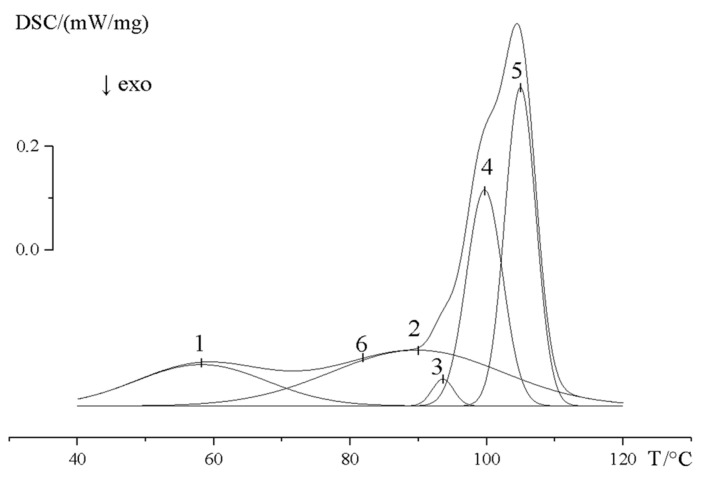
DSC curves of grafted PE copolymer with PS (Δ*p* = 74%) obtained in the presence of 2.5 vol. % DVB. The peak temperatures and peak surface areas were 1—58 °C (10.3 J/g), 2—89.9 °C (18.3 J/g), 3—93.5 °C (1.1 J/g), 4—99.7 °C (15 J/g), and 5—104.5 °C (18.8 J/g), respectively. The total surface area of the crystal phase of PE is 63.64 J/g. The 6 is the glass-transition temperature of PS, corresponding to 83 °C.

Thus, the graft polymerization of St in the presence of DVB leads to the formation of hybrid membranes with a complex heterogeneous structure. This is also confirmed by a thermochemical analysis (Figure 11). Five peaks were detected in the typical DSC curve, which were highlighted using the multiple peak fit (Gaussian) functions of the OriginPro 2017 program. The peak temperatures and peak surface areas were 58 °C (10.3 J/g), 89.9 °C (18.3 J/g), 93.5 °C (1.1 J/g), 99.7 °C (15 J/g), and 104.5 °C (18.8 J/g), respectively. The total surface area of the crystal phase of PE is 63.64 J/g. The degree of crystallinity of the PE crystallites in relation to the melting heat of an ideal PE crystal was estimated to be 4 (1), 6.3 (2), 0.37 (3), 5.1 (4), and 7.4% (5), respectively. We identified transition 6 as the glass-transition temperature of PS, corresponding to 83 °C.

Thus, the dispersion medium is registered: the phases of PE (melting point Tm and degree of crystallinity, Figure 11), the phase of grafted PS distributed in the dispersion medium (glass-transition temperature Tg), and the fragments of cross-linked phases of DVB distributed in both media. In some parts of the PE phase, which probably have an increased content of amorphous fragments, the microphase of the cross-linked PS is surrounded by the crystals (lamellae) of PE (Figure 10b).

**Figure 12:**
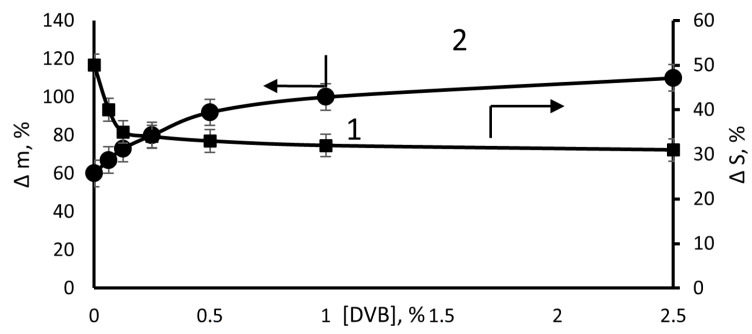
Effect of the DVB concentration in the grafting solution on the change in the surface area (Δ*S*) (1) and weight (Δ*m*) (2) of the PE film with grafted PS during swelling in o-xylene. Δ*p* = 110%. The swelling time was 2 days.

**Figure 13:**
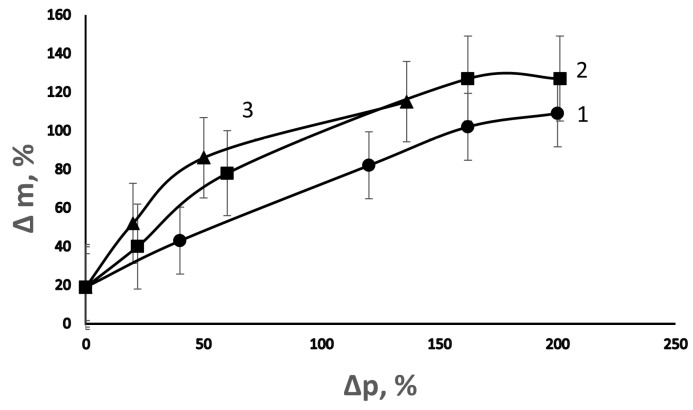
Dependence of the change in the PE film weight during swelling in *o*-xylene on the degree of grafting of PS at various concentrations of DVB in the grafting solution. [DVB], vol. %: 1—0; 2—0.5; and 3—2.5. The swelling time was 3 days.

The membranes obtained in the presence of DVB due to the formation of a three-dimensional structure slightly change their linear dimensions when swelling in organic solvents. However, the solvent sorption does not decrease; in fact, it slightly increases (Figure 12). Apparently, this is due to the formation of a cryptoheterogenic porous structure during grafting. The pore-forming agents are methanol and St. In the films obtained at a high concentration of DVB in the solution of graft polymerization when swelling in o-xylene, the pores are opened and filled with this solvent. They reach a size of several millimeters and are visually distinguishable. Figure 13 shows that the swelling increases both with an increase in the degree of grafting of PS and the content of DVB in the solution of graft polymerization.

Thus, the addition of DVB to the grafting solution allows directional control over a wide range of the distribution of the grafted PS over the thickness of the PE film and the morphology of the modified film.

The self-diffusion coefficients of water measured by the pulsed field gradient technique in the PE film with grafted PS with different DVB contents are shown in Table 1. As shown in our previous papers, water molecules are situated in two parts with different concentrations of sulfonate groups [21,22]. There are two types of water molecules in the films with low DVB contents (Table 1). As shown in Table 1, at DVB contents higher than 1 vol. %, only one type of water molecule is observed in the films. The self-diffusion coefficient of water tends to decrease. The water content λ is one order of magnitude lower than that of an uncross-linked film.

## 4. Conclusions

The effect of divinylbenzene (DVB) on the kinetics of the post-radiation chemical graft polymerization of styrene (St) on the polyethylene (PE) film and its structural and morphological features was investigated. IR transmission, multiply attenuated total internal reflection spectroscopy, X-ray microanalysis, transmission and scanning electron microscopy, DSC, and pulsed field gradient NMR technique were applied. The graft polymerization of St in the presence of DVB leads to the formation of hybrid membranes with a complex heterogeneous structure. In the graft PS phase, microphase particles of cross-linked DVB with fixed interfaces are formed. Extended defects and cracks are formed in the contact zone of the phases. The effect of DVB on the morphology of PE films with graft PS and sulfocationite membranes obtained via the sulfonation of these films is shown. The self-diffusion coefficient of water decreases with increasing DVB content. The membrane humidity is one order of magnitude lower in the films cross-linked with DVB.

## Figures and Tables

**Table 1:** Effect of the DVB concentration on the water content and self-diffusion coefficients of water in the PE film with grafted PS. *D*_1_ and *D*_2_ are the partial self-diffusion coefficients; *p*_1_ and *p*_2_ are the relative parts (populations) of water molecules characterized by self-diffusion coefficients *D*_1_ and *D*_2_; Δ*p* is the degree of grafting (%); *λ* is the number of water molecules per sulfo group.

[DVB], vol. %	Degree of Grafting (∆*p*), %	Water Content *λ*, [H_2_O/SO_3_^−^]	Self-Diffusion Coefficients *D*_1_ and *D*_2_ and Water Populations *p*_1_ and *p*_2_			
			*D*_1_, m^2^/s	*p*_1_	*D*_2_, m^2^/s	*p*_2_
0	142	29.1	6.3 × 10^−10^	0.62	9.0 × 10^−11^	0.38
0.05	91	26	6.1 × 10^−10^	0.85	9.6 × 10^−11^	0.15
1	148	2.6	-	-	6.0 × 10^−11^	-
1	93	3.1	-	-	3.6 × 10^−12^	-
2.5	89	3.2	-	-	2.0 × 10^−11^	-

## Data Availability

Not applicable.
